# Jack Frost Nips at Alabama: Severe Frostbite in the Deep South

**Published:** 2014-07-18

**Authors:** Tony L. Weaver, Derek Robinson, E. Shields Frey

**Affiliations:** Department of General Surgery, Baptist Health Systems, Birmingham, Ala

**Keywords:** frostbite, cold, injury, tissue, salvage

**Figure:**
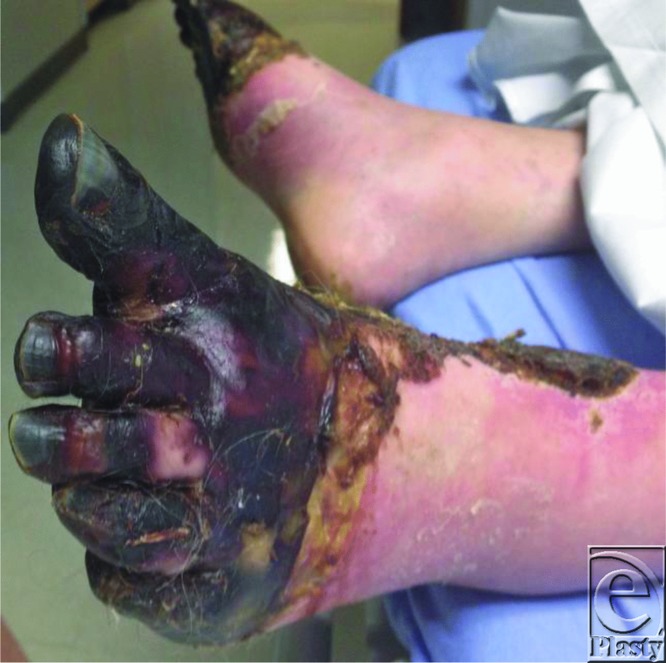


**Figure:**
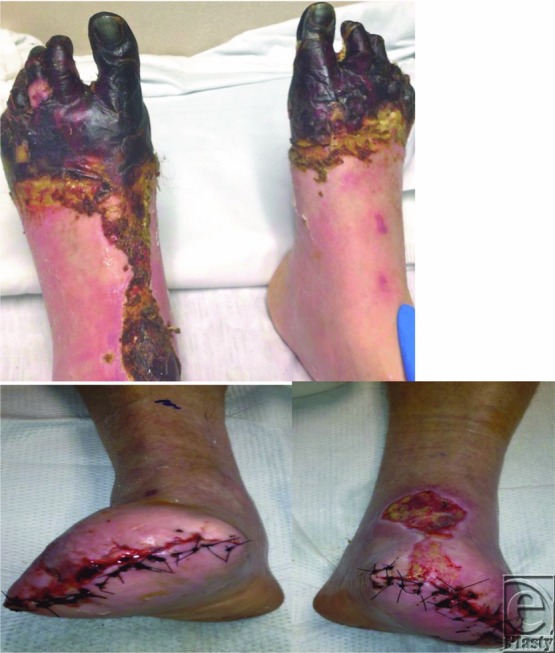


## DESCRIPTION

A 60-year-old man with a medical history of schizoaffective disorder and polysubstance abuse presented in Birmingham, Alabama, with bilateral “black feet.” He reported “a few hours out in the cold” 3 weeks ago. Physical examination revealed forth degree frostbite of the bilateral lower extremities (Figs 1 and 2).

## QUESTIONS

1. **What is the incidence of frostbite and what populations are at risk of developing the condition?**
2. **What is the pathophysiology of frostbite?**
3. **What are the classification systems for frostbite and how is the severity of frostbite determined?**
4. **What are the current recommendations for the surgical management frostbite?**

## DISCUSSION

Jack Frost is a mystical and mythical being who has been referenced in the literary works since the 1800s.^1^ Legend has it that Jack Frost playful nips at your nose, but frostbite portends a worse prognosis. Frostbite is uncommon in Alabama and in the United States in general. The incidence of cold-related injuries and frostbite in the United States is difficult to calculate, because of the lack of a unified reporting system for data collection.^2^ However, multiple studies have evaluated the occurrence in selected populations.^3^ Previous retrospective studies of frostbite injuries have been limited to geographic locations with harsh winter climates and regions of higher elevation.^4^ The most common factors identified in these studies are homelessness, psychiatric illness, vehicular trauma, and drug abuse.^4^ Paradoxically, men aged 30 to 39 years have been found to be at risk. Frostbite has been found to have a predilection to nip at the nose, extremities, ears, and cheeks, and even the corneas.^3^ The extremities are the most susceptible sites; hands/feet account for 90% of all recorded injuries. Other sites include ears, nose, cheeks, and the penis.^5^

Frostbite is a localized injury related to exposure to freezing temperatures.^6^

The contemporary viewpoints identify 2 underlying processes: *Direct Cellular Damage* and *Progressive Dermal Ischemia*.^5^ Direct cellular damage results from the formation of ice crystals within the extracellular space, which results in severe cellular dehydration. As the ice crystals form, membrane disruption, cell destruction, and a phase of microvascular occlusion occurs. The resultant microvascular occlusion is worsened by the body's natural response to cold termed the “Hunting Reaction.” This occurs when the body responds to cold with alternating cycles of vasoconstriction and vasodilation, which then releases inflammatory mediators (thromboxane and prostaglandins) and free radicals into injured tissue which results in further cellular damage and progressive dermal ischemia.^4^

Two classification schemes are used to describe frostbite. Theses systems are based on the depth of injury to the skin and underlying tissue.^4^^,^^6^^,^^7^ The generalized classification of superficial and deep has been associated with a greater efficacy.^7^ Another system assigns a first, second, third, or fourth degree. First degree has hyperemia and edema without blisters. Second degree is hyperemia, edema, and bullous blisters. Third degree has subcutaneous tissue and skin death, small hemorrhagic vesicles, and an eschar, which forms 2 weeks later. Fourth degree is complete necrosis, dry gangrene, and full-thickness tissue loss.^4^^,^^6^^,^^7^ The 2 systems overlap, superficial frostbite can be considered first and second degree and deep frostbite is considered third and fourth.^5^

Surgical management should follow rapid rewarming and medical management. Care should be taken to avoid friction rewarming, which leads to further tissue loss.^4^^,^^6^^,^^7^ The extremity should be rewarmed at 40 to 44 degrees until normal body temperature is achieved over 15 to 30 minutes. Higher temperatures do not warm the injured area faster and cause the warming process to be more painful.^4^^,^^6^^,^^7^ Medical management includes tetanus vaccination, rewarming, wound cleansing, evacuation of blister contents (this releases inflammatory mediators), and pain management. Over a period of 1 to 3 months, frostbite injuries continue to evolve with some recovery of injured tissue.^4^^,^^6^^,^^7^ Surgical management should follow the old adage, “Frostbite in January, amputate in June.” Magnetic resonance imaging and technetium scintigraphy are used to determine the extent of osteomyelitis and tissue loss. Early surgical therapies should be reserved for escharotomies/fasciotomies in the setting of compartment syndrome or debridement in the setting of persistent infection.^4^^,^^7^ Amputation/debridement of infected or necrotic tissue should be conducted once a clear tissue demarcation has been identified.^4^ In studies by Lau et al^8^ and Leonard et al^9^ from Annals of Plastic Surgery, free flaps were used to salvage tissue as well as avascular bone loss following frostbite necrosis. While surgery is not indicated in the initial management it is a mainstay in long-term definitive care.
